# Long Neuro-COVID-19: Current Mechanistic Views and Therapeutic Perspectives

**DOI:** 10.3390/biom14091081

**Published:** 2024-08-28

**Authors:** Anny Slama Schwok, Julien Henri

**Affiliations:** 1 Sorbonne Université, INSERM U938, Biology and Cancer Therapeutics, Centre de Recherche Saint Antoine, Saint Antoine Hospital, 75231 Paris, France; 2 Sorbonne Université, CNRS UMR 7238, Laboratoire de Biologie Computationnelle et Quantitative, Institut de Biologie Paris-Seine, 75005 Paris, France

**Keywords:** long COVID, brain fog, inflammation, mitochondrial dysfunction, immunity, viral reservoirs, nucleoprotein, neurotransmission, substance P, enkephalin

## Abstract

Long-lasting COVID-19 (long COVID) diseases constitute a real life-changing burden for many patients around the globe and, overall, can be considered societal and economic issues. They include a variety of symptoms, such as fatigue, loss of smell (anosmia), and neurological–cognitive sequelae, such as memory loss, anxiety, brain fog, acute encephalitis, and stroke, collectively called long neuro-COVID-19 (long neuro-COVID). They also include cardiopulmonary sequelae, such as myocardial infarction, pulmonary damage, fibrosis, gastrointestinal dysregulation, renal failure, and vascular endothelial dysregulation, and the onset of new diabetes, with each symptom usually being treated individually. The main unmet challenge is to understand the mechanisms of the pathophysiologic sequelae, in particular the neurological symptoms. This mini-review presents the main mechanistic hypotheses considered to explain the multiple long neuro-COVID symptoms, namely immune dysregulation and prolonged inflammation, persistent viral reservoirs, vascular and endothelial dysfunction, and the disruption of the neurotransmitter signaling along various paths. We suggest that the nucleoprotein N of SARS-CoV-2 constitutes a “hub” between the virus and the host inflammation, immunity, and neurotransmission.

## 1. Introduction

Long-lasting COVID-19 diseases (long COVID), defined as symptoms lasting more than two months after the initial SARS-CoV-2 infection [1,2,3], constitute a real burden for many patients around the globe and, overall, can be considered societal and economic issues. About 25% of non-hospitalized COVID-19 patients suffer from long COVID. Age (often young or middle-aged adults), female sex, poor pre-pandemic general and mental health, previous autoimmune conditions, overweight/obesity [4], pollution, and precedent cancers (in particular hematological malignancies) [5] are risk factors for long COVID, suggesting an underestimated importance of immunity and hormonal status, with a possible link to viral load. It is still unclear whether the severity of the initial infection has direct associations with symptoms appearing in the subsequent stages of the disease, but, often, symptoms may appear after a mild or even asymptomatic primary infection [6]. While being of practical clinical importance, the tools for predicting whether a patient will suffer from long COVID or not remain elusive.

In this mini-review, we aim to gather the main current hypotheses underlying long COVID and the current and future therapeutic options for treating long COVID, placing an emphasis on long neuro-COVID. We provide new literature data on the neurological aspects and mechanistic issues, in line with our previous work addressing the altered neuropeptide transmission mediated by the viral nucleoprotein N [7] (see Appendix A).

## 2. Current Hypotheses

**Figure 1:**
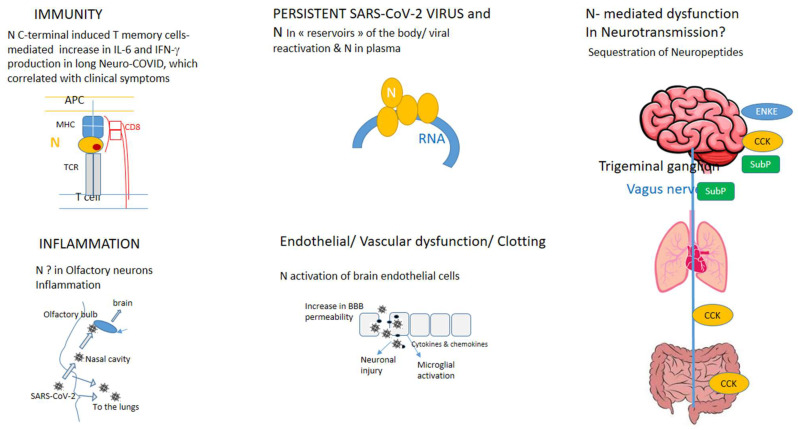
The SARS-CoV-2 nucleoprotein: a hub between immunity [8,9], inflammation [6,7,10,11], including mitochondrial dysfunction, oxidative stress, and brain hypoxia (not depicted) [12,13,14,15,16,17,18,19], viral reservoir(s) [20,21,22,23], and alteration of neurotransmission by neuropeptides [7,15,24,25,26] in long neuro-COVID.

Several hypotheses about what leads to multiple long COVID symptoms have been considered (Figure 1). 

A. **Long COVID diseases could be a consequence of unresolved inflammation and immune dysregulation deriving from the initial infection, with possible long-term functional alterations** [6].

A-1: Inflammation

One hallmark of the initial SARS-CoV-2 infection, which is usually severe, is the dysregulated release of cytokine and chemokines. This “cytokine storm” can be caused by different biochemical processes and may also depend on the lifestyle and environment of the patient, as well as their pre-existing health conditions. The initial pro-inflammatory cytokine production driven by immune cells, such as neutrophils, macrophages, and mast cells, can be amplified by the viral activation of the Aryl hydrocarbon Receptor (AhR). The AhR mediates its effects via alterations in the regulation of mitochondrial function in immune cells [27]. This activation downregulates the endogenous antiviral responses of natural killer cells and memory CD8^+^ T cells. The AhR can also be activated by pollution, depending on hormonal balance, age, and gender. The AhR, associated with tryptophan metabolism, can induce an indoleamine 2,3-dioxygenase (IDO1) IDO1–AhR–IDO1 positive feedback loop, prolonging the activation induced by pathogens [28]. Medications in clinical use, such as dexamethasone, may downregulate both AhR and IDO1 genes. Vitamin D may downregulate the AhR gene, and tocopherol/vitamin E may downregulate the IDO1 gene [28]. Experiments on infected cell lines and in animal studies have shown that AhR antagonists can reduce the viral load and lung inflammation [29]. 

Viral infection also activates a cyclo-oxygenase COX-2 cascade, leading to an enhanced pro-inflammatory cytokine release, and anti-COX NSAID compounds, such as naproxen and indomethacin, have been shown to reduce inflammation by decreasing pro-inflammatory cytokines such as IL-6. Notably, both compounds have also been shown to possess antiviral properties against respiratory viruses and SARS-CoV-2 in animal studies and studies on primary-infected COVID-19 patients [30,31,32,33].

Brain hypoxia, neuronal metabolism, and mitochondria: High levels of oxygen are consumed by neurons for normal brain function. SARS-CoV-2 infection induces hypoxia, increasing glycolysis and impairing mitochondrial oxidative phosphorylation and ATP production [32,34,35,36,37]. In long COVID patients, elevated ferritin levels, indicative of significant iron protein storage dysfunction and low serum iron levels, were associated with brain fog [38]. This is in agreement with the fact that the oxygen binding capacity of severely ill COVID-19 patients has been found to be disturbed in prior studies [38,39]. 

One of the mechanisms through which the SARS-CoV-2 virus hijacks mitochondrial function involves the viral spike protein’s interaction with host mitochondrial monoamine oxidase B, as recently shown in in vitro studies [17]. The mitochondrial ROS and ROS produced by NADPH oxidase-2 (NOX2), known to be activated by viral infection [40], can further lead to fibrosis and platelet aggregation [13], suggesting a potential role for NOX2 inhibitors in long COVID [16,41].

A-2: Immunity

The immune response quantified by the levels of antibodies against SARS-CoV-2 proteins nucleoprotein N and spike S is correlated with the long COVID symptom of fatigue in long COVID patients [10]. In one particular study, the levels of antibodies were lower in patients experiencing fatigue compared to those not experiencing fatigue. In a subsequent study, the researchers found, in a cohort of 101 long COVID patients, that the levels of anti-N antibodies above the median in vaccinated patients constituted an independent predictor for complete remission at follow-up [11], suggesting that the immune response against N may have an impact on the duration for which the fatigue in long COVID is felt. Accordingly, the maintenance of a prolonged inflammatory state that manifests as chronic fatigue is consistent with the involvement of N in cytokine release, as shown in cellular studies and in primary-infected COVID-19 patients [33,42,43,44]. Additionally, N overexpression was associated with the induction of pro-inflammatory M1 macrophage and acute kidney injury in diabetic mice in [45]. Studies proposing that the relatively conserved N should be included in the design of future vaccines have also been published, with such proposals being based on the N-specific CD8^+^ T cell response noted in a clinical study that included 254 long COVID patients [46].

Characteristic T cell changes: T cell immunity is necessary for the host defense against SARS-CoV-2. CD4^+^ and CD8^+^ T cell responses directed against the spike and the nucleoprotein have been found in COVID-19 convalescents [6,47]. The response of N-specific interferon-producing memory CD8^+^ T cells decreases more rapidly in long COVID patients compared to convalescents [6,46]. Long COVID patients suffering from neurological symptoms present a decreased T cell response specific to the spike protein S but an enhanced T cell response against the nucleoprotein N compared to convalescent patients. In particular, a decline in the activation of CD8^+^ memory T cells has been observed [9,46]. The increased severity of cognitive deficits and decreased quality of life markers in these long neuro-COVID patients are positively correlated with IFN-γ production in response to N antigens [9].

T cells express both H1 and H2 histamine receptors, and a treatment with histamine receptor antagonists was reported to attenuate the long COVID symptoms after a mild primary infection in [48]. In addition to T cells, mast cells (MCs) are involved in allergic reactions and secrete histamine and inflammatory cytokines such as IL-6 and TNF-α. The spike protein seems to induce MC and microglia activation in neuro-COVID [49]. MCs are known to interact with neuropeptides that mediate endothelial cell activation, resulting in central nervous system (CNS) inflammatory disorders, as observed mainly in animal studies [25].

A-3: Long Neuro-COVID-19 (Long Neuro-COVID) and neuropeptides

As detailed below, we and others previously identified that defective neurotransmission by some neuropeptides may significantly contribute to neurologic and other symptoms of long COVID [7,26,50] based on cellular and *ex vivo* studies. 

Neuropeptides, including enkephalin, cholecystokinin (CCK), and substance P/tachykinins, are a diverse group of neurotransmitters associated with pain and inflammation, which, together with their receptors, are considered as potential therapeutic targets in human mood disorders and neuropsychiatric disorders in rodent studies and in studies involving addicted patients [7,50,51,52]. The neuropeptide modulation of cortical circuits could modify sensation, decision making, and cognition [52]. Dysfunction in enkephalin’s modulation of neural circuits could contribute to anxiety, depression, and pain regulation and likely lead to decreased protection against neuronal injury via the inhibition of the TLR4/NF-κB signaling pathway, according to animal studies [53].

Proteomic studies showed a role for opioid growth factor receptor signaling in response to SARS-CoV-2 infection: the opioid growth factor-enkephalin suppressed lymphocyte T proliferation, with important implications for immunity (Figure 1) [51]. Proenkephalin was identified as a risk predictor of mortality in COVID-19 patients admitted to an intensive care unit for interstitial pneumonia in [54]. Substance P was involved in the defense of the respiratory tract, as well as in pain, in neurological manifestations of long COVID infection in [26,50] and could be a potential causal factor for long COVID, as deduced from transcriptomic studies using samples of primary-infected COVID-19 patients [24]. Moreover, the dysregulation of substance P levels was observed in Alzheimer’s disease and Parkinson’s disease based on studies using human brain tissues [55]. The roles of synaptic deficits, particularly in some excitatory neurons, and astrocyte impairment in supporting neurotransmission have been reported in studies on severe COVID-19 and in long COVID patients [56]. The neuropeptide CCK was shown to be involved in synaptic transmission based on studies using human brain tissues [57]. Moreover, a derivative of CCK was shown to regulate the mitochondrial function and improve the cognitive deficits in a mouse model of Alzheimer’s disease [15]. 

B. **Persistent viruses found in several tissues and in some lymph nodes could contribute to long-lasting symptoms**, even though no remaining virus can be detected by PCR when extracted from nasal swabs, as shown in the RECOVER cohort [20], in a study of 73 non-hospitalized patients featuring a follow-up for 90 days [58], and in animal studies [59]. These persistent viruses could be considered as “debris” unsuccessfully cleared by a deficient immune system. Additionally, these tissues, including gut tissue, may constitute reservoirs for SARS-CoV-2 and other viruses. Indeed, latent pathogens such as the Epstein–Barr virus and herpes simplex viruses were re-activated in immune-sensitive long COVID patients [23].

In animal models and post-mortem studies, brain invasion by various SARS-CoV-2 variants, leading to brain inflammation and neuron degeneration, has been observed; in one study, different levels of anosmia were observed in the variants. In a 2-year retrospective cohort of patients with long COVID, persistent smell alteration and cognitive impairment were associated with more severe neuro-inflammation and/or with clotting proteins [59]. Olfactory transport and axonal transport via trigeminal nerves, olfactory neurons, the vagus nerve, and/or other nerves are considered important routes of viral brain invasion [21,59,60,61,62]. 

C. **Widespread vascular dysfunction, disruption of the blood–brain barrier (BBB), and/or endothelial coagulopathy** could contribute to microscopic blood clots in the brain and lead to neurological symptoms, even in the absence of a clinically apparent stroke [23]. SARS-CoV-2 infection induces platelet activation, leading to their aggregation and adhesion; consequently, aspirin, which inhibits the COX-1 activation in platelets and thromboxane’s ability to form clots at an endothelial injury site, has been tested in clinical trials in moderately ill patients [22,63]. Platelet activation is also associated with the nitric oxide (NO) pathway. Indeed, in one study, as nitric oxide inhibits platelet adhesion and aggregation, 124 long COVID patients presented lower levels of circulating NO and NO-associated metabolites such as nitrite than 24 patients who had recovered from COVID-19 [64]. In association with the NO pathway and platelet aggregation, recent findings have linked long COVID with reduced serotonin levels, which impacts the activity of the vagus nerve and impairs memory [65]. 

## 3. Insights into the Role of the SARS-CoV-2 Nucleoprotein in COVID-19 and Long COVID

The need to understand the mechanisms underlying the pathophysiologic sequelae of, in particular, the neurological symptoms [66] led us to focus on the nucleoprotein (N) of SARS-CoV-2. We reasoned that the nucleoprotein (N) is primarily required for viral replication and particle assembly through its functional association with the viral ribonucleoprotein complex (RNP complex). N is secondarily involved in the repression of host immunity. N plasma levels are correlated with primary infection severity [43,67], and the detection of N correlates with signs of local inflammation [21,43]. N challenges the human host; therefore, antiviral strategies against N, including ones involving RNA interference and RIG-I-mediated interferon signaling, have been developed to block N’s repression of the host antiviral response [68,69,70,71]. 

Interestingly, N is also associated with additional facets of long-term SARS-CoV-2-specific immune and inflammatory responses [6,9]. In cellular studies, the N protein of SARS-CoV-2 has shown significant mitochondrial localization and an ability to impair the activity of antioxidant enzymes, thus enhancing the mitochondrial ROS levels [19]. Increased oxidative stress is a common mechanism induced by viral pathogens such as SARS-CoV-2 and Influenza A virus, which may enhance their replication at the expense of their host’s metabolism [14,40]. These diseases can develop into acute cognitive impairments similar to brain fog in COVID-19, which has led to the hypothesis that chronic activation of populations of circulating T and B lymphocytes may perpetuate a chronic state of pro-inflammation within the CNS structures by facilitating cross-reactions with viral epitopes [18]. 

Long neuro-COVID patients present wide-ranging alterations in anti-N-specific immune responses, particularly those regarding CD8^+^T cell reactivity, demonstrating the increased production of IL-6 and IFN-γ from N. Moreover, the cognitive and psychiatric clinical measures were correlated with the N-specific IFN-γ production induced by its carboxy-terminal region (amino acids 309–402) in [9]. Interestingly, the C-terminal region of N is a binding site for neuropeptides [7].

In one particular study, N antigens were detected in the blood of patients with encephalopathy and in a patient with neuro-COVID, suggesting unresolved infection and inflammation in some of the non-hospitalized neuro-COVID patients [8]. Finally, N has been associated with endothelial dysfunction [36,72]. 

Therefore, the nucleoprotein of SARS-CoV-2, N, constitutes a molecular hub between the virus and host inflammation, immunity, and neurotransmission (Figure 1).

We previously suggested a mechanism through which N can both function as an actor in viral replication and in host inflammation exacerbation [7]. We showed that N can bind the small neuropeptides involved in pain and in inflammation that act as hormonal mediators or neuronal transmitters, such as substance P and enkephalin. 

On the one hand, the binding of the neuropeptide to N can locally reduce the viral load of the host by impeding RNP assembly. Indeed, in a previous study, the viral load of the infected cells was reduced via treatment with the small molecules bound to N, and the damage induced by the cytokine burst also decreased [33]. Thus, the “sequestration” of the neuropeptides by N could confer to the host additional antiviral protection against the general response induced by the virus. 

On the other hand, however, neuropeptides have been impeded from carrying out their normal functions by the virus, with potential dysfunctions in the normal neurotransmission and/or hormonal signaling between different parts of the body. Potentially, this communication alteration may generate a broad spectrum of symptoms in long neuro-COVID, including brain fog, mood disorders, etc. (Table 1).

**Figure 2:**
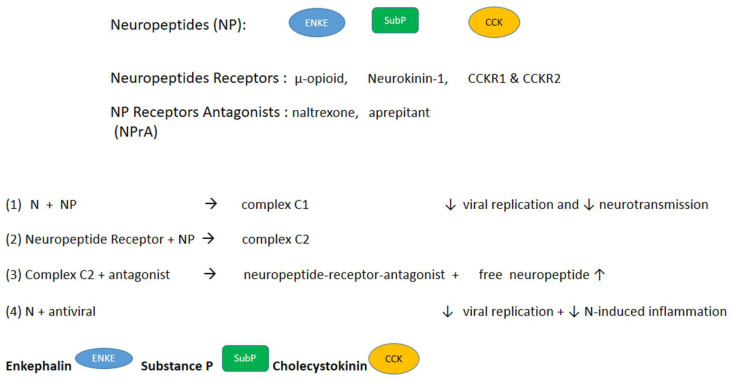
Proposed model. (1) depicts the reversible binding of the neuropeptides (NPs) to N, affecting both replication and neurotransmission; (2) depicts the reversible binding of the neuropeptide to its receptor; in (3), a receptor antagonist can release the free neuropeptide and block the receptor; (4) depicts the direct action of an N-directed antiviral or an indirect action of an RNP-directed antiviral on N.

Here, we propose that the three neuropeptides we identified—substance P, enkephalin, and cholecystokin (CCK)—and their receptors, µ-opioid receptor, neurokinin-1 (NK-1), and CCK receptors, are involved in long neuro-COVID through the perturbations of their neurotransmission, metabolic, and immune functions (Figure 2); the hypothesis we propose is based on recent data from the literature. 

The dysregulation of NP would likely imply abnormal concentration distributions in several parts of the body, in particular in the brain, in the gut, and in the plasma. In line with our hypothesis, antagonists of neuropeptide receptors, namely naltrexone, the antagonist of the µ-opioid receptor, and aprepitant, the antagonist of the NK-1 receptor, showed a clinical benefit in relieving the neurological dysfunction and pain in COVID-19 survivors and long COVID patients (Table 1). We suggest that CCKA and/or CCK2 receptor antagonists such as devazepide or lorglumide could help in long neuro-COVID as well. SARS-CoV-2 infection induces lipid accumulation and the administration of fenofibrate, a PPARα agonist that induces lipid catabolism, reverses metabolic changes, and blocks SARS-CoV-2 replication [34,73], while fenofibrate treatment could result in CCK production [73]. Our hypothesis is also consistent with the imbalance in dopamine transmission thought to be involved in COVID-associated neurodegenerative disease in long COVID patients, although the mechanism(s) may be different [74,75]. Additionally, in another study, the peripheral serotonin concentration was reduced in neuro-COVID, a deficiency that was linked to cognition impairment via reduced vagal signaling [65].

Biochemically speaking, the binding of the neuropeptide to N may reduce the concentration of free peptides, particularly in “reservoirs” where the N concentrations may be higher (as is possibly the case in olfactory neurons/olfactory bulbs/trigeminal nerve lymph nodes) [21,25,52,76]. The addition of an antagonist to the neuropeptide receptor (Figure 2, reaction 3) (for example, naltrexone, an antagonist to the µ-opioid receptor or opioid growth factor receptor [77]) would compete with the binding of the neuropeptide to its receptor (Figure 2, reaction 2), thereby increasing the free concentration of NP, in this example, enkephalin. Indeed, changes in the concentrations of proenkephalin and substance P have been reported in COVID-19 patients: the plasma concentration of proenkephalin is increased in COVID-19 patients [54], and the levels of substance P and its neurokinin receptors have been found to be increased in olfactory neurons, with the latter being proportional to the residual olfaction [76].

An additional/alternative way to restore the level of free neuropeptides via inhibition complex C1 (Figure 2 reaction 1) could involve the administration of an antiviral (Figure 2, reaction 4), assuming that replication is active [33]. The antiviral would inhibit viral replication by directly inhibiting N or the RNP indirectly or inhibiting the cleavage of the polyprotein by inhibiting the SARS-CoV-2 main protease (such as Paxlovid). Quantitative proteomic studies analyzing the concentration of neuropeptides and the nucleoprotein in the serum and in other fluids, as well as in post-mortem brains, the gastrointestinal tract, and lymph nodes, could help to establish a predictive model and shed light on the already available therapeutical options. 

The neuropeptides could modulate each other, with their dysfunctional neurotransmission being potentially cross-exacerbated in long neuro-COVID. Cholecystokinin (CCK) is the most effective endogenous anti-opioid peptide. The binding of CCK to CCK receptors reduced the binding affinity of opioids (morphin, heroin, and beta-endorphins) to their opioid receptors. The neuropeptides enkephalin and CCK exhibited a strikingly similar distribution within many areas of the CNS and within the nociceptive centers, including part of the spinal cord and the brain (Figure 1), and they are functionally antagonistic in a number of circumstances [78,79]. Interestingly, opioid treatment may potentially increase the neurotropism of the SARS-CoV-2 infection, with possible links to gut microbial dysbiosis [80]. CCK is also a satiety hormone and a transmitter that mediates sugar/nutrient sensing, conveying information between the gut and the brain via the vagus nerve [35,37] and linking metabolism, neurotransmission/signaling, and, possibly, viral persistence/inflammation. 

The consequences of broken neurotransmission could be profound changes in the energy/metabolism in the brain. As mentioned above, a SARS-CoV-2 infection induced hypoxia, increased glycolysis and lipid accumulation [15,23]. Many links between some neuropeptides and mitochondrial respiration/hypoxia/ischemia have been reported in the literature. For example, neuronal survival, achieved through the improved mitochondrial respiration facilitated by proenkephalin treatment under hypoxic conditions, was reported in a rat model of epileptogenesis [12]. The treatment of brain endothelial cells with an enkephalin derivative improved the mitochondrial function under hypoxia and relieved ischemia/reperfusion injury [51]. Moreover, a CCK derivative regulated the mitochondrial function and improved the cognitive deficits in a mouse model of Alzheimer’s disease [15]. 

Therefore, the “sequestration” of these neuropeptides in mitochondrial-associated N could further enhance mitochondrial dysfunction, avoiding their protecting effects on metabolism and against hypoxia. This would, in turn, further enhance the mitochondrial ROS formation and overcome the inhibition of replication by the neuropeptide, provided that the N–neuropeptide binding (complex C2, reaction 2) remains stable in the mitochondria. It is likely that the “sequestration” by N would decrease the pain-relieving effects of opioid peptides. 

**Table 1:** Description of N ligands [7] and their normal interactions/function(s) and related long neuro-COVID-19 symptoms.

Hormone/Neuropeptide	Target	Receptor	Antagonist	Symptom(s)	References
Cholecystokinin (CCK)	Gut and brain and vagus nerve	CCKR1 and R2, CBRs receptors		- Pancreas dysfunction as CCK is a satiety hormone - Synaptic deficits: CCK is involved in synaptic transmission via the activation of muscarinic acetylcholine receptors	[15,51,57,73,78,79]
Met/Proenkephalin	Brain/ inhibition of replication	Opioid receptors	Naltrexone	Pain reduction, increased gut dysbiosis, neurodegenerescence	[7,12,44,53,54,78,81,82,83,84]
Substance P	Olfactory neurons Trigeminal nerve lymph node	Neurokinin receptor-1 (NK-1R)	Aprepitant	Pain transmission, headache, brain fog, depression, thromboembolism, pro-inflammatory effects, and viral latency (although the latter requires further study).	[24,50,76,85,86]
Folate/heme/iron	Heart, blood hemoglobin	Folate receptor, iron metabolism, Fe storage ferritin		Brain fog, shortness of breath, ferroptosis, fibrosis	[7,38,39,87]

## 4. Conclusions

Long COVID is a life-changing disease for afflicted patients. In this paper, we aimed to present the current hypotheses and develop a biochemical model for long neuro-COVID based on the altered neurotransmission of selected neuropeptides, namely cholestocytokinin, enkephalin, and substance P, between the brain and the gut through the vagus nerve and the CNS (Figure 1; [7]). These neuropeptides were trapped by the viral protein N, which modified the distribution of the free neuropeptides and their binding to their respective receptors. We cannot rule out the contributions of other viral proteins, such as the spike protein S, as both N and S have been found in the brains of COVID-19 patients and are associated with microvascular and immune cells’ activation [60,88]. The administration of the receptors’ antagonists would increase the concentration of circulating neuropeptides and likely restore neurotransmission. As a consequence, a reduction in symptoms such as brain fog, anxiety, gut dysbiosis, and, possibly, fatigue can be expected. We cannot rule out the possible involvement of additional neuropeptides/neurotransmitters such as dopamine and their binding to N as N has a large C-terminal cavity, possibly further impacting neuro-COVID patients [74,75]. The model encompasses the hypothesis of incomplete viral clearance/the build-up of viral reservoirs and of immune/inflammation perturbations in long COVID, including those directly caused via microglia or indirectly caused via mast cell activation (Figure 1) [25,44,89]. It would be interesting to test whether the intracellular cellular reservoirs in infected cells such as macrophages could be addressed by manipulating the cellular mechanisms of, for example, autophagy or cell death signaling, as has been proposed for HIV [90]. The administration of antivirals in cases of incomplete viral clearance from “reservoirs” would likely be beneficial to some long neuro-COVID patients [8]. The neurological symptoms and encephalopathies are not specific to neuro-COVID patients. Similar symptoms have been observed in some cases of infection via other viruses [91], namely Influenza A in the 1918 pandemic, herpes simplex, HIV, Chikungunya, Dengue, and Zika virus, although the tropism of coronaviruses is not specific to the CNS. Identifying the common mechanisms in order to avoid the virus perverting the host response and possibly remaining in the reservoirs is an important task that should be carried out in order to protect humankind against future viral infections and identify adequate repurposed drugs/treatments.

## Appendix A

**Importance of the article for readership:**

Long COVID diseases are a societal burden and an everyday pain for patients as they often suffer from multiple symptoms. This review paper aims to present the current mechanistic hypotheses underlying these symptoms, with an emphasis on long neuro-COVID-19.

**Statement of concrete aims and questions:**

The goal is to offer therapeutic options and, ultimately, to provide tools that could help to monitor the development of long COVID for clinics. For each of the hypotheses presented, we aim to discuss the available therapeutic options or the putative options we or others suggest.

**Description of Literature search:**

A search of the PubMed database was performed using keywords, including terms such as “SARS-CoV-2” or “coronavirus” or “COVID-19”; “Long COVID” or “PASC (post- acute sequelae of COVID-19)”; “inflammation” or “cytokine storm” or “IL-6”; “viral reservoirs” or “viral reactivation”; “pain”, “brain fog”, “fatigue”; “nucleoprotein” or “nucleocapsid”; “spike protein”; “ROS” or “oxidative stress” or “mitochondrial dysfunction” or “NADPH oxidase”; and “neurotransmission” or “neuropeptides.”

Key Statements Are All Supported by Literature References

**Scientific reasoning:**

Along with the common pathways described, we bring together the multiple roles of the SARS-CoV-2 nucleoprotein N that represent, in our view, a hub between viral replication, host inflammation and mitochondrial dysfunction, immunity, and neurotransmission, as summarized in Figure 1.

**Presentation of the data:**

We further describe a testable biochemical model to explain our hypotheses of (at least) some mechanisms of long neuro-COVID-19 associated with N, depicted in Figure 2, and gather the known properties of the neuropeptides listed in Table 1.
